# Clinical features of genetically characterized types of hereditary angioedema with normal C1 inhibitor: a systematic review of qualitative evidence

**DOI:** 10.1186/s13023-020-01570-x

**Published:** 2020-10-15

**Authors:** Konrad Bork, Thomas Machnig, Karin Wulff, Guenther Witzke, Subhransu Prusty, Jochen Hardt

**Affiliations:** 1 Department of Dermatology, University Medical Center, Johannes Gutenberg University, Langenbeckstr. 1, 55131 Mainz, Germany; 2 CSL Behring GmbH, Marburg, Germany; 3 University Medicine, Ernst Moritz Arndt University, Greifswald, Germany; 4 Department of Medical Psychology and Medical Sociology, Johannes Gutenberg University, Mainz, Germany

**Keywords:** Hereditary angioedema, Hereditary angioedema with normal C1 inhibitor, HAE with a specific mutation in the *F12* gene, HAE with a specific mutation in the plasminogen gene, HAE with a specific mutation in the angiopoietin-1 gene, HAE with a specific mutation in the kininogen-1 gene, HAE with a specific mutation in the myoferlin gene, Clinical features, Asphyxia, Tongue swelling

## Abstract

**Background:**

Hereditary angioedema (HAE) with normal C1 inhibitor (C1-INH) (HAEnCI) is associated with skin swellings, abdominal attacks, and the risk of asphyxia due to upper airway obstruction. Several different gene mutations linked to the HAE phenotype have been identified. Our aim was to qualitatively assess and describe the clinical differentiators of these genetically identified HAEnCI types. To achieve this, we performed a systematic literature review of patients with angioedema symptoms and a genetically confirmed diagnosis of an HAEnCI type.

**Results:**

A systematic literature search, conducted in March 2020, returned 132 records, 43 of which describe patients with symptoms of angioedema and a genetically confirmed diagnosis of an HAEnCI type. Overall, this included 602 patient cases from 220 families. HAEnCI with a mutation in the coagulation factor XII gene (*F12*) (HAE-FXII) was diagnosed in 446 patients from 185 families (male:female ratio = 1:10). Estrogens (oral contraceptives, hormonal replacement therapy, and pregnancy) negatively impacted the course of disease in most female patients (252 of 277). Asphyxia occurred in 2 of 446 patients. On-demand and/or long-term prophylaxis treatment included C1-INH concentrates, icatibant, progestins, and tranexamic acid. HAEnCI with a specific mutation in the plasminogen gene (HAE-PLG) was diagnosed in 146 patients from 33 families (male:female ratio = 1:3). Estrogens had a negative influence on the course of disease in the minority of female patients (14 of 62). Tongue swelling was an important clinical feature. Asphyxia occurred in 3 of 146 patients. On-demand treatment with icatibant and C1-INH concentrate and long-term prophylaxis with progestins and tranexamic acid were effective. HAEnCI with a specific mutation in the angiopoietin-1 gene (HAE-ANGPT1) was diagnosed in 4 patients from 1 family and HAEnCI with a specific mutation in the kininogen-1 gene (HAE-KNG1) in 6 patients from 1 family.

**Conclusions:**

A number of clinical differentiators for the different types of HAEnCI have been identified which may support clinicians to narrow down the correct diagnosis of HAEnCI prior to genetic testing and thereby guide appropriate treatment and management decisions. However, confirmation of the causative gene mutation by genetic testing will always be required.

## Background

Angioedema is a hallmark sign of various hereditary and acquired conditions. It is characterized by recurrent localized and self-limited edema episodes in various organs. Clinical symptoms include swelling of the skin and tongue, abdominal pain attacks, as well as pharyngeal and laryngeal edema that can lead to death by asphyxia [1, 2].

The most common type of hereditary angioedema (HAE) is the result of impaired C1 inhibitor (C1-INH) activity (HAE-C1-INH) due to protein deficiency (type I) or dysfunction (type II) and was identified in 1963 by Virginia Donaldson [3]. In 1987, the link to mutations in the *SERPING1* gene was revealed by Southern blot analysis using *SERPING1*-specific gene probes [4].

In 2000, a novel type of HAE was identified which was linked with normal C1-INH activity in plasma and classified as "HAE with normal C1-INH" (HAEnCI) or "HAE type III" [5, 6]. A breakthrough in the understanding of HAEnCI came with the discovery of several different mutations in genes involved in kallikrein-kinin system activation, each of which being linked to the HAE phenotype.

In 2006, previously unknown mutations in exon 9 of the coagulation factor XII (FXII) gene (*F12*) were identified in families with HAEnCI, resulting in the term HAE-FXII [7]. However, since not all HAEnCI patients harbored an *F12* gene mutation, it was assumed that there must be mutations in further genes resulting in HAEnCI [7, 8].

**Fig. 1:**
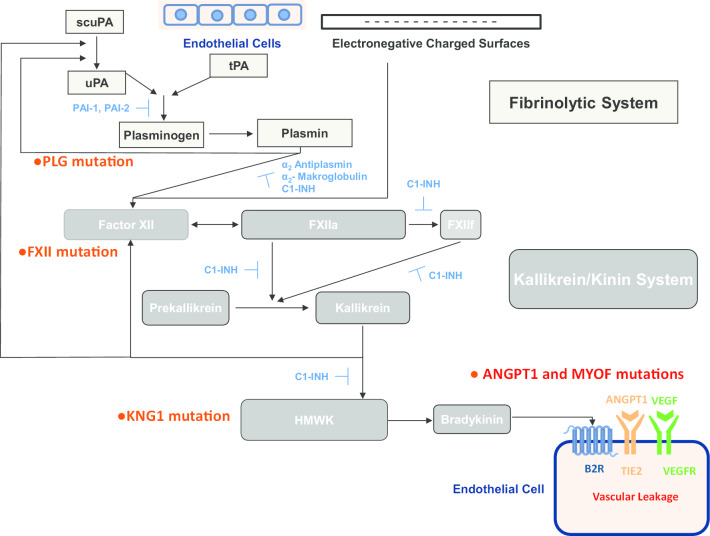
Mutations linked to various types of HAEnCI and its impact on the various proteases and protease inhibitors of the fibrinolytic and kallikrein-kinin systems. ANGPT1 = angiopoietin-1 gene, B2R = bradykinin-2 receptor, C1-INH = C1 esterase inhibitor, FXII = coagulation factor XII gene, HMWK = high molecular weight kininogen, KNG1 = kininogen-1 gene*,* MYOF = myoferlin gene, PAI = plasminogen activator inhibitor, PLG = plasminogen gene, TIE2 = tyrosine-protein kinase, tPA = tissue plasminogen activator, scuPA = single-chain urokinase-type plasminogen activator, uPA = urokinase-type plasminogen activator, VEGF = vascular endothelial growth factor

Subsequently, in large families with 3 or more generations, further HAEnCI-linked mutations were identified by linkage analysis in genes involved in the formation of bradykinin: the plasminogen (*PLG*), angiopoietin-1 (*ANGPT1*), kininogen-1 (*KNG1*), and myoferlin (*MYOF*) genes [9–12] (Fig. 1).

Based on these findings, 4 additional HAEnCI types were defined: HAE with a specific mutation in the *PLG* gene (HAE-PLG), HAE with a specific mutation in the *ANGPT1* gene (HAE-ANGPT1), HAE with a specific mutation in the *KNG1* gene (HAE-KNG1), and HAE with a specific mutation in the *MYOF* gene (HAE-Myoferlin).

In many families with HAEnCl, the genetic background of the disease is still unknown (referred to as "HAE-unknown") [8, 13]. Table 1 summarizes the various types of HAE identified until now.

**Table 1:** Types of hereditary angioedema

HAE type	Gene	Nucleotide change	Protein change	Chromosome	First described by	Methods used
HAE-C1-INH	*SERPING1*	Numerous	Numerous	11	Stoppa-Lyonnet et al. [4]	Southern blot analysis, linkage analysis
HAE-FXII	*F12*	c.983C > A	p.T328K	5	Dewald and Bork [7]	Candidate gene, Sanger sequencing, linkage analysis
		c.983C > G	p.T328R	5	Dewald and Bork [7]	Candidate gene, Sanger sequencing, linkage analysis
		c.971_1018 + 24del72	Indel	5	Bork et al. [13]	Sanger sequencing, linkage analysis
		c.892_909dup	dup p.298_303	5	Kiss et al. [14]	Sanger sequencing, linkage analysis
HAE-PLG	*PLG*	c.988A > G	p.K330E	6	Bork et al. [10]	WES, linkage analysis, Sanger sequencing
HAE-ANGPT1	*ANGPT1*	c.807G > T	p.A119S	8	Bafunno et al. [9]	WES, linkage analysis
HAE-KNG1	*KNG1*	c.1136 T > A	p.M379K	3	Bork et al. [11]	WES, linkage analysis, Sanger sequencing
HAE-Myoferlin	*MYOF*	c.651G > T	p.R217S	10	Ariano et al. [12]	WES, linkage analysis
HAE-unknown	ni^a^	na	na	na	na	na

*ANGPT1* = angiopoietin-1 gene, bp = base pairs, C1-INH = C1 esterase inhibitor, del = deletion, dup = duplication, *F12* = coagulation factor XII gene, FXII = coagulation factor XII protein, HAE = hereditary angioedema, HAE-ANGPT1 = HAE with a specific angiopoietin-1 gene mutation, HAE-C1-INH = HAE due to C1-INH deficiency, HAE-FXII = HAE with a specific coagulation FXII gene mutation, HAE-KNG1 = HAE with a specific kininogen-1 gene mutation, HAE-Myoferlin = HAE with a specific myoferlin gene mutation, HAE-PLG = HAE with a specific plasminogen gene mutation, *KNG1* = kininogen-1 gene, na = not applicable, ni = not identified, *MYOF* = myoferlin gene, *PLG* = plasminogen gene, WES = whole exome sequencing

^a^By definition

For the novel types of HAEnCI described until March 2020—namely HAE-FXII, HAE-PLG, HAE-ANGPT1, and HAE-KNG1—we aimed to summarize and analyze the differences in clinical characteristics such as demographics, trigger factors, location of attacks, and response to various treatments. Therefore, we performed a systematic literature review of these genetically defined HAEnCI types neglecting the cases with HAE-unknown and the cases of individuals carrying 1 of the known HAEnCI mutations but without clinical manifestation of the disease. Since HAE-Myoferlin was described shortly after our literature search was conducted, we mention it here in the interest of completeness but have not included the patient cases for further analysis.

## Methods

### Systematic literature search

A systematic literature search was conducted in the US National Library of Medicine, National Institutes of Health database PubMed for a period from 1 January 2006 to 1 March 2020, which was limited to publications in English. We supplemented our search by hand searching the bibliographies and abstracts of key papers.

We identified 706 publications that met our electronic search strategy: "complement c1 inhibitor protein" (Medical subject headings [MeSH] Terms) and "angioedema, hereditary" (MeSH Terms). Of these, 132 records described HAEnCI patients. Since the nature of data was inappropriate for meta-analysis, a qualitative synthesis was performed. All publications which reported on unique patient cases with symptomatic angioedema and a genetically confirmed HAEnCI were included in our data analysis.

**Fig. 2:**
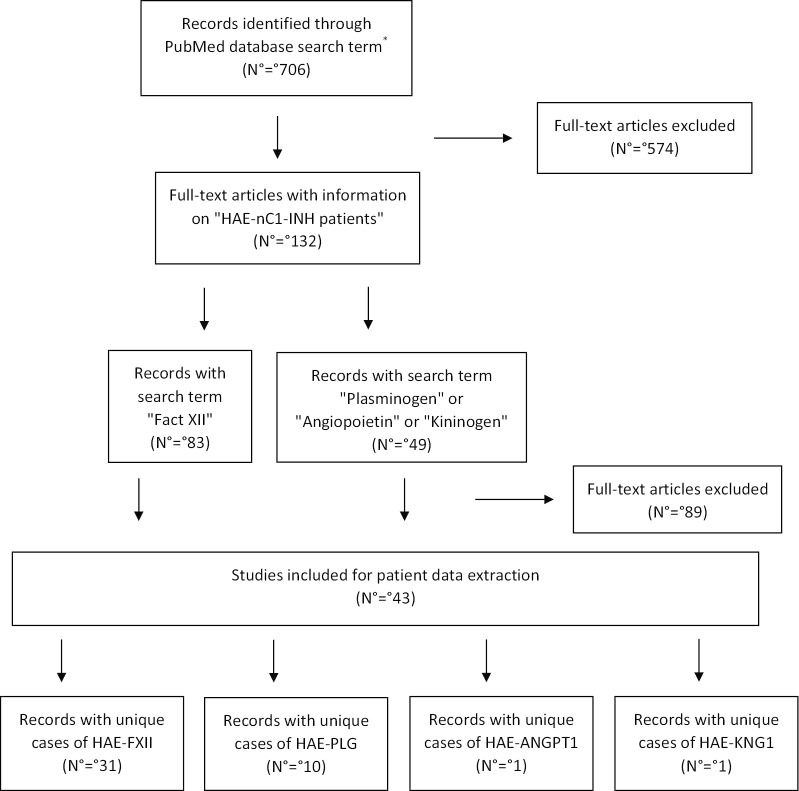
QUOROM flow-chart shows the number of publications screened and included in the systematic review for qualitative evidence. HAE-ANGPT1 = hereditary angioedema with a specific mutations in the angiopoietin-1 gene, HAE-FXII = hereditary angioedema with specific mutations in the factor XII gene, HAE-KNG-1 = hereditary angioedema with a specific mutation in the kininogen-1 gene*,* HAE-PLG = hereditary angioedema with a specific mutation in the plasminogen gene, HMWK = high molecular weight kininogen, HAEnCI = hereditary angioedema with normal C1 esterase inhibitor, MeSH = medical subject headings, N = number of records. *("complement c1 inhibitor protein"[MeSH Term] AND ("angioedema, hereditary" [MeSH Term]) search for a period from 1 January 2006 to 1 March 2020

Excluded were cases on patients/families without an HAEnCI-specific mutation or cases of asymptomatic carriers. Patients described in more than 1 publication were counted only once. Publications identified as being potentially relevant were retrieved in full-text format and screened. A QUOROM flow-chart showing the number of publications screened and those included for our qualitative synthesis is depicted in Fig. 2.

## Results

The literature search results and selection of articles for data extraction are summarized in Tables 2 and 3. The search returned 43 unique records that were eligible for patient data extraction. Overall, 602 affected HAEnCI patients coming from 220 families were identified.

**Table 2:** Cases of clinically affected patients/families with HAE-FXII (gender, age of onset, impact of estrogen)

Described by	Nucleotide change	Type of mutation	Protein change	No. of families	No. of patients	No. of males	No. of females	Age of onset (years)	No. of females impacted by estrogens			Origin of patients
									Clear impact	No impact	Not exposed	
Bork et al. [13]^a^	c.983C > A	Missense	p.Thr328Lys	23	69	1	68	20.3 ± 9.2 (mean ± SD)	48	10	10	Germany, Turkey, Brazil
	c.983C > G	Missense	p.Thr328Arg									
	c.971_1018 + 24del72	Deletion	Indel									
Cichon et al. [16]	c.983C > A	Missense	p.Thr328Lys	1	4	0	4	nr	nr	nr	nr	France
Martin et al. [17]	c.983C > A	Missense	p.Thr328Lys	1	4	2	2	nr	2	0	0	France
Bouillet et al. [18]	c.983C > A	Missense	p.Thr328Lys	1	2	0	2	19 (from 1/2 pts)	2	0	0	France
Bell et al. [19]	c.983C > A	Missense	p.Thr328Lys	1	1	0	1	20	0	1	0	Australia
Duan et al. [20]^b^	c.983C > A	Missense	p.Thr328Lys	1	7	0	7	nr	7	0	0	Italy
Prieto et al. [21]	c.983C > A	Missense	p.Thr328Lys	1	4	0	4	35 (from 1/4 pts)	4	0	0	Spain
Hentges et al. [22]	c.983C > A	Missense	p.Thr328Lys	1	1	0	1	nr	1	0	0	Luxembourg
Nagy et al. [23]	c.983C > A	Missense	p.Thr328Lys	1	3	0	3	nr	2	1	0	Italy
Picone et al. [24]	c.983C > A	Missense	p.Thr328Lys	2	5	0	5	nr	3	2	0	France
Baeza et al. [25]	c.983C > A	Missense	p.Thr328Lys	1	3	0	3	20 (from 1/3 pts)	3	0	0	Morroco
Marcos et al. [26]	c.983C > A	Missense	p.Thr328Lys	13	29	3	26	23.9 (14–55) (mean[range])	25	1	0	Spain
Kiss et al. [15]	c.892_909dup	Duplication	p.Pro298_Pro303dup	1	1	0	1	35	0	1	0	Hungary
Gomez-Traseira et al. [27]	c.983C > A	Missense	p.Thr328Lys	1	5	0	5	22.8 (mean)	5	0	0	Spain
Charignon et al. [28]	c.983C > A	Missense	p.Thr328Lys	40	80	8	72	24.3 (mean; from 69/80 pts)^e^	nr	nr	nr	France, Spain
Moreno et al. [29]	c.983C > A	Missense	p.Thr328Lys	4	8	1	7	13.9 (mean)^e^	7	0	0	Brazil
Mansi et al. [30]	c.983C > A	Missense	p.Thr328Lys	3	3	nr	nr	nr	nr	nr	nr	Italy
Stieber et al. [31]	c.983C > A	Missense	p.Thr328Lys	1	12	0	12	27 (mean; from 7/12 pts)^e^	4	nr	nr	Brazil
Moreno et al. [32]	c.983C > A	Missense	p.Thr328Lys	1	1	0	1	16	1	0	0	Brazil
Deroux et al. [33]	c.983C > A	Missense	p.Thr328Lys	24	37	1	36	21. ± 7.27 (mean ± SD)^e^	32	4	0	France
Grumach et al. [34]	c.983C > A	Missense	p.Thr328Lys	2	14	4	10	27.7 (mean; from 12/14 pts)	8	2	0	Brazil
Piñero-Saavedra et al. [35]	c.983C > A	Missense	p.Thr328Lys	9	24	3	21	19.9 (mean; from 16/21 f); 61.5 (mean; from 2/3 m)	19	nr	nr	Spain
Bork et al. [36]^c^	c.983C > A	Missense	p.Thr328Lys	1	4	0	4	nr	nr	nr	nr	Germany
Veronez et al. [37]	c.971_1018 + 24del72	Deletion	Indel	1	1	1	0	68	0	0	1^f^	Brazil
Veronez et al. [38]	c.983C > A	Missense	p.Thr328Lys	41	101	15	86	21.1 ± 11.7 (2–68) (mean ± SD [range])	59	nr	nr	Brazil
Bova et al. [39]^d^	c.983C > A	Missense	p.Thr328Lys	9	23	0	23	21 (5–76) (median [range])	20	3	0	Italy

C1-INH = C1 esterase inhibitor, F = females, FXII = coagulation factor XII, Indel = insertion/deletion, Lys = lysine, M = males, No. = Number; nr = not reported, Pro = proline, pts = patients, SD = standard deviation, Thr = threonine

^a^Article also includes all patients described in Dewald and Bork [7], Bork et al. [8], Bork et al. [14], Bork et al. [40], and Bork et al. [41]

^b^This family has been described by Binkley and Davis [5]

^c^Besides the here mentioned family, this article includes patients published in [13]

^d^This article includes a patient series published by Firinu et al. [42]

^e^Recalculated

^f^1 male patient exposed to an ACE inhibitor [37]

**Table 3:** Mutation, demographics, and gender of patients with HAE-PLG, HAE-ANGPT1, and HAE-KNG1

Described by	Nucleotide change	Type of mutation	Protein change	No. of families	No. of patients	No. of males	No. of females	Age of onset (years)	No. of females impacted by estrogens			Origin of patients
									Clear impact	No impact	Not exposed	
HAE-PLG												
Bork et al. [10]^a^	c.988A > G	Missense	p.Lys330Glu	13	60	13	47	30.5 ± 15.5 (5–72) (mean ± SD [range])	6	37	4	Germany
Germenis et al. [54]	c.988A > G	Missense	p.Lys330Glu	3	4	0	4	40,41,75 (from 3/4 pts)	1	2	1	Greece, Bulgaria, Spain
Belbezier et al. [55]	c.988A > G	Missense	p.Lys330Glu	3	8	2	6	23 (6–64) (mean [range])	2	4	0	France
Yakushiji et al. [56]	c.988A > G	Missense	p.Lys330Glu	2	4	1	3	47.3 (mean)	0	3	0	Japan
Bodian et al. [49]	c.988A > G	Missense	p.Lys330Glu	1	1	0	1	21	0	1	0	USA
Recke et al. [50]	c.988A > G	Missense	p.Lys330Glu	1	6	3	3	20–60 (range)	nr	nr	nr	Germany
Bork et al. [51]	c.988A > G	Missense	p.Lys330Glu	1	12	6	6	27 (16–56) (mean [range]; from 7/12 pts)	5	1	0	Germany
Bork et al. [52]^b^	c.988A > G	Missense	p.Lys330Glu	9	51	nr	nr	nr	nr	nr	nr	Germany
HAE-ANGPT1												
Bafunno et al. [9]	c.807G > T	Missense	p.Ala119Ser	1	4	0	4	“Second decade”	0	4	0	Italy
HAE-KNG1												
Bork et al. [11]	c.1136 T > A	Missense	p.Met379Lys	1	6	1	5	35 ± 16.2 (17–55) (mean ± SD [range])^c^	2	3	0	Germany

Glu = glutamic acid, HAE-ANGPT1 = HAE with a specific angiopoietin-1 gene mutation, HAE-FXII = HAE with a specific coagulation FXII gene mutation, HAE-KNG1 = HAE with a specific kininogen-1 gene mutation, HAE-PLG = HAE with a specific plasminogen gene mutation, Lys = lysine, Met = methionine, No. = number, Pts = patients, nr = not reported, SD = standard deviation

^a^The same families were published later by Dewald [53]

^b^This article furthermore contains the patients published by Bork et al. [10]

^c^Recalculated

HAE-FXII was identified in 446 patients from 185 different families based on 31 records; 5 records were excluded due to double reporting of cases.

HAE-PLG was identified in 146 patients from 33 families based on 10 records; 2 records were excluded due to double reporting.

For HAE-ANGPT1 and HAE-KNG1, 1 record was identified each (Table 3): 4 cases from 1 family with HAE-ANGPT1 and 6 cases from 1 family with HAE-KNG1.

Only recently, after the cut-off for the research period of our literature review, a novel HAEnCI type was described in an Italian family of 3 women with angioedema symptoms [12]. The symptoms were limited to the face, lips, and oral mucosa. Since a MYOF-217S gain-of-function variant was identified as the genetic cause, leading to an abnormal myoferlin protein involved in VEGF signal transduction, this type was named HAE-Myoferlin.

### Hereditary angioedema with a specific mutation in the F12 gene

Since 2006, when HAE-FXII-specific mutations were first described [7], further affected patients/families have been identified using genetic standard methods for linkage analysis (Sanger sequencing). These include large families of 2 or more generations that were previously diagnosed with HAEnCI of unknown cause. Based on observations of our Angioedema Outpatient Service in Mainz (Germany), we estimate the prevalence of HAE-FXII in Germany at 1:400,000 [13].

Following the first description of 2 different missense mutations in the *F12* gene, 2 further mutations were identified: a *F12* gene deletion and a *F12* gene duplication. Up to now, a total of 185 families with HAE-FXII have been described, including 179 families with a Thr328Lys mutation, 2 with a Thr328Arg mutation, 3 with a *F12* deletion mutation, and 1 family with a duplication mutation. All 4 HAE-specific mutations are in the *F12* gene region coding for the proline-rich region of the FXII protein (Table 2). HAE-FXII with missense mutations c.983C > A (p.Thr328Lys) and c.983C > G (p.Thr328Arg) in the *F12* gene was first described in 2006 in patients from 6 unrelated families [7]. Currently, 181 families with 440 HAE patients with these gene mutations are reported in literature (Table 2).

HAE-FXII with a large deletion mutation of 72 base pairs on the *F12* gene (c.971_1018 + 24del72) was first described in 2011 in a family of Turkish origin, including 2 sisters and their symptom-free father [14]. The deleted region includes codon p.Thr328 and therefore also the position of the two previously identified missense mutations: p.Thr328Lys and p.Thr328Arg [7]. It is a partial exon 9/intron 9 deletion, causing a loss of 48 base pairs of exon 9 (coding amino acids 324 to 340, including the authentic donor splice site) and of 24 base pairs of intron 9) [14]. In 2014, a second family (from Turkey) [41] and in 2018, a third family with an affected pair of siblings (from South America) [37] were reported.

HAE-FXII with a duplication mutation (c.892_909dup) in the *F12* gene was reported in a 37-year-old female patient and her daughter [15]. Screening *of F12* for this duplication revealed that 4 family members carried the mutation.

### Inheritance, gender, penetrance, transmission

Among the 185 different families with 446 patients with HAE-FXII, the overall male to female ratio was about 1:10. In a cohort with 42 patients from Brazil the ratio was 1:6 whereas in the remaining patients the male to female ratio was 1:13 [38]. The reason for the higher prevalence of male patients in Brazil is unknown.

HAE-FXII is inherited as an autosomal-dominant trait with incomplete penetrance. In 1 patient series, overall penetrance of HAE-FXII was 66.3%: Males had a penetrance of 4.0% and females a penetrance of 86.1% [13].

Individuals with homozygous genotype of Thr328Lys mutations have been described and appear to have a more severe phenotype [34].

### Patient origin, age of onset, clinical symptomatology, and attack triggers

To date most patients with HAE-FXII were observed in Brazil, France, Spain, and Germany (Table 2). In addition, reports come from various other countries including Australia and Morocco.

Symptom manifestation was usually around the age of 20 (range 1–65 years) [13]. Estrogens (oral contraceptives [OC], menstruation, pregnancy, and hormone replacement therapy [HRT]) often play an important role as triggering or aggravating factors in HAE-FXII [5, 8, 13, 26, 33, 35, 36, 38, 39, 42, 43]. In 17 of 35 female patients, the first HAE attacks coincided with the intake of OCs. Other females experienced their first symptoms during pregnancy or after start of HRT [8]. Further triggers include angiotensin converting enzyme (ACE) inhibitors [36, 37, 39, 42]. A few patients have been reported to have received ACE inhibitors without any influence on their disease [39]. Aggravation of symptoms with angiotensin-1 receptor blocker has only been observed in 1 case [8].

For 277 female patients with HAE-FXII, the impact of estrogens on the course of disease was described. Estrogens triggered or exacerbated HAE symptoms in 252 (91.0%) and had no impact in 25 (9.0%). Similarly, it was reported in a case series that HAE-FXII started or worsened in 90% of females taking OC, while 10% did not notice any negative effect [13]. In 58 females with 92 pregnancies, symptom onset or exacerbation was reported for 43 pregnancies, no influence for 42, and an improvement of HAE-FXII symptoms for 7 pregnancies. While in some females with HAE-FXII, clinical symptoms were limited to periods of OC intake, HRT, and pregnancies ("estrogen-dependent angioedema"), 1 patient reported the opposite and became symptom-free during all 3 of her pregnancies [8]. Clinical symptoms seem to be independent of the type of *F12* gene mutation, and include skin swellings, abdominal pain attacks, tongue swellings, and life-threatening laryngeal edema [8, 38]. Among the 446 patients, 2 cases of asphyxia due to edema have been reported [13], resulting in an asphyxia rate of 1:225.

Most skin swellings were on the face or just on the lips, but other locations such as extremities, genitals, neck, and ears were affected as well.

In 2007, hemorrhages into skin swellings on the face or extremities were observed in HAE-FXII. They started 1–2 days after onset of the swelling and were not associated with anticoagulant treatment or bleeding disorders [44]. Subsequently, 15 more patients with bruising or ecchymosis at the site of skin swelling or other sites were reported [35, 42]. In 1 patient, bruising preceded abdominal pain attacks [38]. In total, skin bleedings were reported in 17 of 446 (3.8%) patients with HAE-FXII.

Erythema marginatum, a gyrated erythematous rash on the chest or other parts of the trunk that often precedes or accompanies edema attacks in HAE-C1-INH, was not reported in HAE-FXII patients [8]. Prodromes such as unusual fatigue, chest discomfort, and palpitation have been observed in a few patients [35].

The frequency of attacks varied greatly between patients; while some remained symptom-free most of the time, others suffered from almost daily attacks. There is a report of a female patient who had her first and only swelling at age 67 while another 78-year-old woman experienced over 1000 angioedema attacks in 64 symptomatic years [8].

### Treatment experiences

Current therapeutic strategies focus on reducing disease burden and improvement of quality of life based on 2 treatment approaches: on-demand treatment of acute attacks and prophylactic treatment, for both short and long-term use.

Since no specific treatments are approved for HAE-FXII, current treatment strategies include similar medications and treatment principles as have shown to be effective in patients with HAE-C1-INH [45].

The following section describes treatment strategies that were applied in patients with HAE-FXII. The first step of the treatment algorithm involves the strict avoidance of exacerbating factors and the discontinuation of certain co-medications, mainly estrogen-containing OCs and HRT, as well as ACE inhibitors [36].

For on-demand treatment, C1-INH concentrates, either plasma-derived or recombinant, icatibant, or ecallantide were most commonly used [26, 35, 36, 39, 46]. The mean duration of facial swelling observed in 11 females was markedly reduced by treatment with plasma-derived C1-INH (Berinert; CSL Behring, Marburg, Germany) compared with no treatment (28 versus 64 h [h]) [36]. Other cases of good treatment response to C1-INH have also been reported [38, 39]. Icatibant was reported by Bouillet et al. to be effective for a severe abdominal HAE-FXII attack, with onset of symptom improvement within 30 min and complete resolution by 1 h [47]. Similarly, Veronez et al. showed a marked reduction in duration and intensity of attacks in 9 females taking icatibant [38]. Bova et al. reported good response in 7 of 9 patients who received icatibant for HAE-FXII attacks, compared with 2 non-responders [39].

For short-term prophylaxis prior to dental, surgical procedures, or other invasive medical interventions as well as for deliveries, it has been reported that intravenous plasma-derived C1-INH effectively prevented attacks [35]. No attacks occurred when C1-INH was used for short-term prophylaxis in 3 patients for esophago-gastro-duodenoscopy, 3 patients for dental procedures, and 1 patient for bronchoscopy [39].

For long-term prophylactic treatment, progestins, tranexamic acid (TXA), C1-INH, and danazol were used [8, 35, 36]. There are several reports on good treatment response to long-term prophylaxis with progestins. For 16 females, an effective switch from estrogen-containing oral contraceptives to progestins was shown (99.8% mean reduction of attack frequency) [36]. Likewise, Veronez et al. reported partial or complete control in 27 females with long-term prophylaxis with progestins [38] and a patient who took a desogestrel-containing pill daily remained symptom-free for 2.5 years [35]. TXA was also shown to be effective for long-term prophylaxis in patients with HAE-FXII. Nearly complete control was achieved in 4 female patients [36], partial or complete control in 30 patients [38], and partial control in 8 patients [26, 39]. When used temporarily in 3 female patients, 1500 mg daily TXA resulted in reduced frequency and severity of symptoms [35].

C1-INH was shown to be effective for long-term prophylaxis during pregnancy, achieving complete control in 1 patient and partial control in another patient [48], and almost complete control of HAE-FXII in 2 members of a family [39]. In 1 patient, partial attack control was reported on concomitant prophylaxis with TXA and C1-INH [39].

Improvement was also reported for long-term prophylaxis with attenuated androgens in 3 females using danazol with a complete response [36], and 7 females with danazol and 4 patients using oxandrolone with complete or partial control [38]. Piñero-Saavedra et al. reported that 1 patient who received danazol (600 mg daily) for 1 year did not experience improvement in symptoms until she stopped taking estrogen-containing OCs [35].

### Hereditary angioedema with a specific mutation in the plasminogen gene

In 2018, a previously unknown mutation in the *PLG* gene was identified by whole exome sequencing (WES). It was transmitted in an autosomal-dominant manner and was linked to clinical symptoms of HAE. The genetic alteration was a c.988A > G missense mutation leading to an amino acid exchange p.Lys330Glu (K330E), which affected the kringle 3 domain of the PLG protein. Thus, a novel type of HAEnCI was proposed, since a total of 14 patients from 4 families harbored the PLG gene mutation, while an HAE-specific *F12* gene mutation was missing [10]. In the same report, 9 of 38 index patients from 38 families, which were classified as having HAEnCI of unknown genetic background, were re-diagnosed by Sanger sequencing with HAE-PLG. Thereby, 13 German families with the PLG mutation were identified [10]. Subsequently, more cases of HAE-PLG were identified in various European countries, the United States, and Japan [49–56]. Thus, a total of 146 patients from 33 families were reported (Table 3).

### Inheritance, gender

The mutation is heterozygous, transmitted as an autosomal-dominant trait, and linked with the HAE phenotype across pedigrees. Information about gender is available for 95 HAE-PLG patients, of which 25 (26.3%) were male and 70 (73.7%) were female. Hence, the gender ratio was 1:3 (male:female).

### Patient origin, age of onset, clinical symptomatology, and attack triggers

Most patients were observed in Germany and France; others were identified in Japan, the United States, and various European countries (Table 3).

In the first German patient series, which described 60 patients from 13 families with HAE-PLG, the mean age (± standard deviation [SD]) of onset of clinical symptoms was 30.5 ± 15.5 years (range 5 to 72 years) (Table 3).

The age of onset of clinical symptoms may be a distinguishing feature. While symptoms of HAE-C1-INH and HAE-FXII usually manifest during the first 2 decades of life, they may start nearly at any age in HAE-PLG. This is an important observation for counseling asymptomatic members of families with HAE-PLG, since the risk of developing the disease remains life-long.

Of the 60 German patients, 47 patients experienced recurrent swelling of the face, lips, and an average of 80.7 tongue swellings before the correct diagnosis was established. In 11 patients, recurrent tongue swellings were the only symptom, with a range of 1–150 swellings at the time of diagnosis. In 23 patients, 8.7% (331 of 3795) of all reported tongue swellings were associated with dyspnea, voice changes, and imminent asphyxia. In 1 female patient, a total of 160 episodes of tongue swellings were described before she died from asphyxia due to an upper airway obstruction [10]. Death by asphyxia was observed in 2 other female patients [10, 50]. Hence, for the 146 patients reviewed here, an asphyxia rate of 1:50 was determined.

In a case series from France, 10 patients were described with a median annual number of attacks of 5 and a median attack duration of 2 days [55]. OCs, ACE inhibitors, and psychological stress have been observed to trigger or exacerbate attacks in HAE-PLG patients [10, 50, 51, 54–56].

In contrast to HAE-FXII, only a minority of females (14.0%; 6 of 43 females) with HAE-PLG were reported to experience initial clinical symptoms after OC intake and there were no reports of an onset during pregnancy [10]. As shown in Table 3, information on the impact of estrogen was available in 62 females: a negative impact on the course of HAE was described by 14 (22.6%) and no impact by 48 (77.4%) patients. It is noteworthy that, across multiple case series and reports, estrogens appeared to play a consistently small physiological role in triggering attacks in HAE-PLG compared with HAE-FXII.

### Treatment experience

Treatment experience with on-demand treatments was recently reported for a larger case series of 111 patients [52]. Of these, 13 patients were treated with icatibant for 201 acute swelling attacks and 12 patients were treated with plasma-derived C1-INH for 74 acute swelling attacks. In an indirect comparison, icatibant appeared to provide a better treatment effect on attack duration than C1-INH, and a higher proportion of patients responded to treatment. The mean duration (± SD) of icatibant treated attacks (4.3 ± 2.6 h) was significantly shorter than that of the previous 149 untreated attacks (44.7 ± 28.6 h; *p* < 0.0001) within the same patients. The mean duration (± SD) of attacks treated with C1-INH (31.5 ± 8.6 h) was significantly shorter than that of the previous 129 untreated attacks (48.2 ± 32.5 h; *p* < 0.0001) [52]. Furthermore, 2 HAE-PLG patients of French origin reported improvement in attacks upon icatibant treatment [55].

Long-term prophylaxis, evaluated by attack frequency before and after treatment, appeared to be more effective in 3 patients treated with TXA (93.9% mean reduction of attack frequency) compared with 6 patients treated with progestins (46.3%), and compared with 3 patients treated with danazol (83.3%) [52].

TXA was also effective in 2 French patients and in 1 Japanese female patient with HAE-PLG [55, 56].

### Hereditary angioedema with a specific mutation in the angiopoietin-1 gene

In 2018, a novel type of HAEnCI, classified as HAE-ANGPT1, was identified by WES in 2 patients from 1 family, and was linked to a mutation in exon 2 of the *ANGPT-1* gene (c.807G > T, p.Ala119Ser [A119S]) [9]. The mutation was confirmed to be present in 2 other symptomatic family members but absent in 7 asymptomatic family members of 3 generations by Sanger sequencing. The mutation was inherited in an autosomal-dominant manner and is presumed to impair the interaction with its membrane receptor, leading to increased plasma leakage of bradykinin.

Patients with HAE-ANGPT1 had a history of recurrent angioedema since their second decade of life without any identifiable trigger, with a frequency of 2 attacks per year and a median attack duration of 33 h (range 1–2 days).

Attacks were primarily located on the face, lips, oral mucosa, and abdomen. Trigger factors were reported in a minority of HAE attacks only and included mechanical stimuli such as minor trauma and prolonged pressure. Two patients responded to prophylaxis with oral TXA with reduced number and severity of attacks. Patients did not respond to antihistamines and corticosteroids, when used for acute attacks or as prophylaxis [9].

### Hereditary angioedema with a specific mutation in the kininogen-1 gene

In 2019, in a large family with hitherto HAE-unknown, an HAE-linked mutation was identified by WES in exon 10 of the *KNG1* gene (c.1136 T > A, p.M379K [Met379Lys]) [11]. Pedigree analysis revealed that clinical symptoms of HAE occurred in 3 generations and co-segregated with the *KNG1* mutation (c.1136 T > A) in all analyzed patients (HAE-KNG1). The mutation is inherited in a dominant manner.

The mutation was found in the cleavage region for kinins, including bradykinin. Bradykinin is the presumed mediator for the symptoms of HAE. All investigated symptomatic family members carried the described *KNG1* gene mutation. The mutation was not present in any of the 38 index HAEnCI patients without any specific mutation in the *F12*, *PLG*, or *ANGPT1* genes.

Patients had facial, tongue, hand, and feet swellings as well as abdominal attacks. In 1 patient, only abdominal attacks were observed. The mean age (± SD) at onset was 35 ± 16.2 years (range 17–55 years). Triggering factors included pressure, OCs, and pregnancies. In 1 patient, symptom relief was reported for 2 facial attacks, 30 min after treatment with 1000 IU C1-INH concentrate. Corticosteroids and antihistamines were ineffective [11].

## Discussion

To the best of our knowledge, this is the first systematic literature review of case series of patients diagnosed with 1 of the genetically defined types of HAEnCI described until March 2020. Including the most recent HAEnCI type described by Ariano et al. 2020, 8 different HAE-specific mutations were identified up to now: 4 are located in the *F12* gene and the other 4 in the *PLG, ANGPT-1, KNG-1*, and *MYOF* genes (Table 1). All types of HAEnCI mutations seem to affect the pathways of the fibrinolytic and kallikrein-kinin system at different levels, leading to bradykinin-mediated vascular leakage and angioedema formation via activation of the bradykinin B2 receptor (Fig. 1).

Our present systematic review assesses the evidence of clinical features from all cases and case series published until March 2020, which can be expected to provide a more comprehensive insight into the different HAEnCI types than single reports. Indeed, we identified clinical differentiators of the different genetic types of HAEnCI (Table 4). All of them are quantitative in nature as they are more prevalent in 1 HAE type than in the others.

**Table 4:** Clinical differentiators between HAE-FXII, HAE-PLG, HAE-ANGPT1, and HAE-KNG1

	HAE-FXII N = 446	HAE-PLG N = 146	HAE-ANGPT1 N = 4	HAE-KNG1 N = 6
Genetics				
Specific mutations serving for identification of HAE type	c.983C > A [7]	c.988A > G [10]	c.807G > T [9]	c. 1136 T > A [11]
	c.983C > G [7]			
	c.971_1018 + 24del72 [14]			
	c.892_909dup [15]			
Gender ratio	Clear female predominance			
Male	39	25	0	1
Female	404	70	4	5
Not reported	3	51	0	0
Ratio (M:F)	1:10	1:3	0:4^a^	1:5^a^
Penetrance	High penetrance in females (86%), low in males (4%) [13]	nr	nr	nr
Clinical features				
Average age of onset (years)	20 [13]	31 [10]	nr	35 [11]
Location of attacks	Small number of tongue swellings	Large number of tongue swellings [10]	No tongue swellings	Small number of tongue swellings
Hemorrhages (into the skin swellings or bruising preceding abdominal attacks)	17/446 (3.8%) patients; possibly a unique feature; not observed in other genetically defined types	0	0	0
Clinical course	Some patients have symptoms exclusively when exposed to estrogens (OC, HRT, pregnancy) "estrogen-dependent angioedema"	Some patients have exclusively tongue swellings [10]; considered to be a unique clinical feature	nr	nr
Deaths by asphyxia (asphyxia rate)	2 (1:225)	3 (1:50)	0	0
Estrogen impact				
Clear impact	252	14	0	2
No impact	25	48	4	3
Pathogenesis				
Protein change	Introduction of new cleavage sites for plasmin in FXII [57]; cleavage of mutant FXII by thrombin and FXIIa [58]	Change of the kringle 3 domain of PLG [10]	Impairment of ANGPT1 [9]	Change of bradykinin cleavage from HMWK [11]

ANGPT1 = angiopoietin, FXII = coagulation factor XII, HAE-ANGPT1 = HAE with a specific angiopoietin-1 gene mutation, HAE-FXII = HAE with a specific FXII gene mutation, HAE-KNG1 = HAE with a specific kininogen-1 gene mutation, HAE-PLG = HAE with a plasminogen gene mutation, HMWK = high molecular weight kininogen, M:F = male:female, N = number of patients, nr = not reported, PLG = plasminogen

^a^1 family only

The clinical differentiators identified were: First, the aggravating effect of estrogens (OC, HRT, pregnancy) observed in HAE-FXII, which was much more pronounced than in other types of HAEnCI. Second, hemorrhage at the swelling site, which up to now, has been reported in HAE-FXII but not in HAEnCI with other mutations. And third, the high frequency of tongue swellings in HAE-PLG, which not only occurred more frequently than in other types of HAEnCI but was often reported as the only type of swelling with no other clinical manifestation.

Interestingly, erythema marginatum, often observed in HAE-C1-INH, was not reported in any of the types of HAEnCI.

These features may serve to delineate the phenotypes of the different types of HAEnCI, opening the field for future research, e.g. regarding the influence of additional pathogenic gene mutations or functional gene polymorphisms on the clinical characteristics of these new HAEnCI types.

HAE-FXII and HAE-PLG have been described in 185 families and 33 families. In contrast, HAE-ANGPT1 and HAE-KNG1 have so far only been described in 1 family each, indicating that the prevalence of the genetically identified types of HAEnCI is highly variable. Since the different HAE types can be diagnosed only by genetic testing, the prevalence rates depend on the number of genetic tests for these recently described HAE types. Test frequency may vary for different reasons, including its availability, access to genetic testing, and awareness of the types of HAEnCI.

A plausible explanation for the uneven distribution of the different types of HAEnCI across the global regions is that the highest prevalence is reported at locations where the founding patient originated, most likely in Europe. The spread to other regions is determined by various socio-economic factors and therefore it may take several generations before these patients are found in other regions of the world.

Current treatment strategies for HAEnCI rely on those already approved for HAE-C1-INH. Attempts have been made to customize treatment strategies in HAE-FXII and HAE-PLG based on the molecular pathways involved in the pathogenesis [36, 52]. However, the exact pathogenic mechanism has yet to be elucidated, despite encouraging findings in HAE-FXII [57–59].

Our scope to identify further differentiating features of the various HAEnCI types was limited due to incomplete reporting on certain aspects, such as prevalence, penetrance, and long-term outcomes.

As discussed above, our findings show that several clinical differentiators exist between the genetically identified types of HAEnCI. The underlying mutations hamper with the biochemical function of different proteins of the kallikrein-kinin and fibrinolytic system pathways (Fig. 1). We conclude that these different gene mutations lead to different biochemical disturbances, resulting in different clinical features. Therefore, we suggest each HAEnCI type to be considered as a distinct disease entity.

## Conclusion

This systematic literature review of 602 reported cases with the genetically determined types of HAEnCI, coming from 220 families, shows that there are several clinical differentiators for the different types. They include specific clinical characteristics and symptoms which are found to be more common in some types of HAEnCI than in others. A targeted diagnosis of HAE using certain clinical characteristics can be helpful for differential diagnosis but cannot replace a genetic work-up of cases to identify the specific genetic mutation. This is considered important to better define specific management and treatment strategies for angioedema patients diagnosed with these new diseases.

## Data Availability

The datasets used and/or analyzed during the current study are available from the corresponding author on reasonable request.

## Abbreviations

ANGPT1: Angiopoietin-1
ACE: Angiotensin converting enzyme
C1-INH: C1 inhibitor
F12: Coagulation factor XII gene
FXII: Coagulation factor XII
HAE: Hereditary angioedema
HAE-ANGPT1: HAE with a specific mutation in the *ANGPT1*
HAE-C1-INH: HAE due to C1-INH deficiency
HAEnCI: HAE with normal C1-INH
HAE-FXII: HAE with a specific mutation in the *F12* gene
HAE-KNG1: HAE with a specific mutation in the *KNG1* gene
HAE-Myoferlin: HAE with a specific mutation in the *MYOF* gene
HAE-PLG: HAE with a specific mutation in the *PLG* gene
HRT: Hormone replacement therapy
h: Hours
KNG1: Kininogen-1
MeSH: Medical subject headings
MYOF: Myoferlin gene
OCs: Oral contraceptives
PLG: Plasminogen
SD: Standard deviation
TXA: Tranexamic acid
WES: Whole exome sequencing

## References

[1] Angioedema BK (2013). Immunol Allergy Clin North Am.

[2] Cicardi M, Aberer W, Banerji A, Bas M, Bernstein JA, Bork K (2014). Classification, diagnosis, and approach to treatment for angioedema: consensus report from the Hereditary Angioedema International Working Group. Allergy.

[3] Donaldson VH, Evans RR (1963). A biochemical abnormality in hereditary angioneurotic edema: absence of serum inhibitor of C1-esterase. Am J Med.

[4] Stoppa-Lyonnet D, Tosi M, Laurent J, Sobel A, Lagrue G, Meo T (1987). Altered C1 inhibitor genes in type I hereditary angioedema. N Engl J Med.

[5] Binkley KE, Davis A (2000). Clinical, biochemical, and genetic characterization of a novel estrogen-dependent inherited form of angioedema. J Allergy Clin Immunol.

[6] Bork K, Barnstedt SE, Koch P, Traupe H (2000). Hereditary angioedema with normal C1-inhibitor activity in women. Lancet.

[7] Dewald G, Bork K (2006). Missense mutations in the coagulation factor XII (Hageman factor) gene in hereditary angioedema with normal C1 inhibitor. Biochem Biophys Res Commun.

[8] Bork K, Wulff K, Hardt J, Witzke G, Staubach P (2009). Hereditary angioedema caused by missense mutations in the factor XII gene: clinical features, trigger factors, and therapy. J Allergy Clin Immunol.

[9] Bafunno V, Firinu D, D’Apolito M, Cordisco G, Loffredo S, Leccese A (2018). Mutation of the angiopoietin-1 gene (ANGPT1) associates with a new type of hereditary angioedema. J Allergy Clin Immunol.

[10] Bork K, Wulff K, Steinmuller-Magin L, Braenne I, Staubach-Renz P, Witzke G (2018). Hereditary angioedema with a mutation in the plasminogen gene. Allergy.

[11] Bork K, Wulff K, Rossmann H, Steinmuller-Magin L, Braenne I, Witzke G (2019). Hereditary angioedema cosegregating with a novel kininogen 1 gene mutation changing the N-terminal cleavage site of bradykinin. Allergy.

[12] Ariano A, D’Apolito M, Bova M, Bellanti F, Loffredo S, D’Andrea G (2020). A myoferlin gain-of-function variant associates with a new type of hereditary angioedema. Allergy.

[13] Bork K, Wulff K, Witzke G, Hardt J (2015). Hereditary angioedema with normal C1-INH with versus without specific F12 gene mutations. Allergy.

[14] Bork K, Wulff K, Meinke P, Wagner N, Hardt J, Witzke G (2011). A novel mutation in the coagulation factor 12 gene in subjects with hereditary angioedema and normal C1-inhibitor. Clin Immunol.

[15] Kiss N, Barabas E, Varnai K, Halasz A, Varga LA, Prohaszka Z (2013). Novel duplication in the F12 gene in a patient with recurrent angioedema. Clin Immunol.

[16] Cichon S, Martin L, Hennies HC, Muller F, Van Driessche K, Karpushova A (2006). Increased activity of coagulation factor XII (Hageman factor) causes hereditary angioedema type III. Am J Hum Genet.

[17] Martin L, Raison-Peyron N, Nothen MM, Cichon S, Drouet C (2007). Hereditary angioedema with normal C1 inhibitor gene in a family with affected women and men is associated with the p.Thr328Lys mutation in the F12 gene. J Allergy Clin Immunol.

[18] Bouillet L, Ponard D, Rousset H, Cichon S, Drouet C (2007). A case of hereditary angio-oedema type III presenting with C1-inhibitor cleavage and a missense mutation in the F12 gene. Br J Dermatol.

[19] Bell CG, Kwan E, Nolan RC, Baumgart KW (2008). First molecular confirmation of an Australian case of type III hereditary angioedema. Pathology.

[20] Duan QL, Binkley K, Rouleau GA (2009). Genetic analysis of Factor XII and bradykinin catabolic enzymes in a family with estrogen-dependent inherited angioedema. J Allergy Clin Immunol.

[21] Prieto A, Tornero P, Rubio M, Fernandez-Cruz E, Rodriguez-Sainz C (2009). Missense mutation Thr309Lys in the coagulation factor XII gene in a Spanish family with hereditary angioedema type III. Allergy.

[22] Hentges F, Hilger C, Kohnen M, Gilson G (2009). Angioedema and estrogen-dependent angioedema with activation of the contact system. J Allergy Clin Immunol.

[23] Nagy N, Greaves MW, Tanaka A, McGrath JA, Grattan CE (2009). Recurrent European missense mutation in the F12 gene in a British family with type III hereditary angioedema. J Dermatol Sci.

[24] Picone O, Donnadieu AC, Brivet FG, Boyer-Neumann C, Fremeaux-Bacchi V, Frydman R (2010). Obstetrical complications and outcome in two families with hereditary angioedema due to mutation in the F12 gene. Obstet Gynecol Int.

[25] Baeza ML, Rodriguez-Marco A, Prieto A, Rodriguez-Sainz C, Zubeldia JM, Rubio M (2011). Factor XII gene missense mutation Thr328Lys in an Arab family with hereditary angioedema type III. Allergy.

[26] Marcos C, Lopez Lera A, Varela S, Linares T, Alvarez-Eire MG, Lopez-Trascasa M (2012). Clinical, biochemical, and genetic characterization of type III hereditary angioedema in 13 Northwest Spanish families. Ann Allergy Asthma Immunol.

[27] Gomez-Traseira C, Lopez Lera A, Drouet C, Lopez-Trascasa M, Perez-Fernandez E, Favier B (2013). Hereditary angioedema caused by the p.Thr309Lys mutation in the F12 gene: a multifactorial disease. J Allergy Clin Immunol..

[28] Charignon D, Ghannam A, Defendi F, Ponard D, Monnier N, Lopez-Trascasa M, Launay D (2014). Hereditary angioedema with F12 mutation: factors modifying the clinical phenotype. Allergy.

[29] Moreno AS, Valle SO, Levy S, Franca AT, Serpa FS, Arcuri HA (2015). Coagulation factor XII gene mutation in brazilian families with hereditary angioedema with normal C1 inhibitor. Int Arch Allergy Immunol.

[30] Mansi M, Zanichelli A, Coerezza A, Suffritti C, Wu MA, Vacchini R (2015). Presentation, diagnosis and treatment of angioedema without wheals: a retrospective analysis of a cohort of 1058 patients. J Intern Med.

[31] Stieber C, Grumach AS, Cordeiro E, Constantino-Silva RN, Barth S, Hoffmann P (2015). First report of a FXII gene mutation in a Brazilian family with hereditary angio-oedema with normal C1 inhibitor. Br J Dermatol.

[32] Moreno AS, Maia LS, Palhas PB, Dias MM, Muglia VF, Castelli EC (2016). Genetic analysis as a practical tool for diagnosis of hereditary angioedema with normal C1 inhibitor: a case report. J Investig Allergol Clin Immunol.

[33] Deroux A, Boccon-Gibod I, Fain O, Pralong P, Ollivier Y, Pagnier A (2016). Hereditary angioedema with normal C1 inhibitor and factor XII mutation: a series of 57 patients from the French National Center of Reference for Angioedema. Clin Exp Immunol.

[34] Grumach AS, Stieber C, Veronez CL, Cagini N, Constantino-Silva RN, Cordeiro E (2016). Homozygosity for a factor XII mutation in one female and one male patient with hereditary angio-oedema. Allergy.

[35] Piñero-Saavedra M, Gonzalez-Quevedo T, Saenz de San Pedro B, Alcaraz C, Bobadilla-Gonzalez P, Fernandez-Vieira L (2016). Hereditary angioedema with F12 mutation: Clinical features and enzyme polymorphisms in 9 Southwestern Spanish families. Ann Allergy Asthma Immunol.

[36] Bork K, Wulff K, Witzke G, Hardt J (2017). Treatment for hereditary angioedema with normal C1-INH and specific mutations in the F12 gene (HAE-FXII). Allergy.

[37] Veronez CL, Serpa FS, Pesquero JB (2017). A rare mutation in the F12 gene in a patient with ACE inhibitor-induced angioedema. Ann Allergy Asthma Immunol.

[38] Veronez CL, Moreno AS, Constantino-Silva RN, Maia LSM, Ferriani MPL, Castro FFM (2018). Hereditary angioedema with normal C1 inhibitor and F12 mutations in 42 Brazilian families. J Allergy Clin Immunol Pract.

[39] Bova M, Suffritti C, Bafunno V, Loffredo S, Cordisco G, Del Giacco S (2019). Impaired control of the contact system in hereditary angioedema with normal C1-inhibitor. Allergy.

[40] Bork K, Wulff K, Witzke G, Stanger C, Lohse P, Hardt J (2013). Antihistamine-resistant angioedema in women with negative family history: estrogens and F12 gene mutations. Am J Med.

[41] Bork K, Wulff K, Hardt J, Witzke G, Lohse P (2014). Characterization of a partial exon 9/intron 9 deletion in the coagulation factor XII gene (F12) detected in 2 Turkish families with hereditary angioedema and normal C1 inhibitor. Haemophilia.

[42] Firinu D, Bafunno V, Vecchione G, Barca MP, Manconi PE, Santacroce R (2015). Characterization of patients with angioedema without wheals: the importance of F12 gene screening. Clin Immunol.

[43] Binkley KE, Davis AE (2003). Estrogen-dependent inherited angioedema. Transfus Apher Sci.

[44] Bork K, Gul D, Hardt J, Dewald G (2007). Hereditary angioedema with normal C1 inhibitor: clinical symptoms and course. Am J Med.

[45] Zuraw BL, Bork K, Binkley KE, Banerji A, Christiansen SC, Castaldo A (2012). Hereditary angioedema with normal C1 inhibitor function: consensus of an international expert panel. Allergy Asthma Proc.

[46] Bouillet L, Boccon-Gibod I, Gompel A, Floccard B, Martin L, Blanchard-Delaunay C (2017). Hereditary angioedema with normal C1 inhibitor: clinical characteristics and treatment response with plasma-derived human C1 inhibitor concentrate (Berinert(I)) in a French cohort. Eur J Dermatol.

[47] Bouillet L, Boccon-Gibod I, Ponard D, Drouet C, Cesbron JY, Dumestre-Perard C (2009). Bradykinin receptor 2 antagonist (icatibant) for hereditary angioedema type III attacks. Ann Allergy Asthma Immunol.

[48] Garcia JFB, Takejima P, Veronez CL, Aun MV, Motta AA, Kalil J (2018). Use of pdC1-INH concentrate for long-term prophylaxis during pregnancy in hereditary angioedema with normal C1-INH. J Allergy Clin Immunol Pract.

[49] Bodian DL, Vilboux T, Hauser NS (2019). Genotype-first analysis of a generally healthy population cohort supports genetic testing for diagnosis of hereditary angioedema of unknown cause. Allergy Asthma Clin Immunol.

[50] Recke A, Massalme EG, Jappe U, Steinmuller-Magin L, Schmidt J, Hellenbroich Y (2019). Identification of the recently described plasminogen gene mutation p.Lys330Glu in a family from Northern Germany with hereditary angioedema. Clin Transl Allergy.

[51] Bork K, Zibat A, Ferrari DM, Wollnik B, Schon MP, Wulff K (2020). Hereditary angioedema in a single family with specific mutations in both plasminogen and SERPING1 genes. J Dtsch Dermatol Ges.

[52] Bork K, Wulff K, Witzke G, Machnig T, Hardt J (2020). Treatment of patients with hereditary angioedema with the c.988A>G (p.Lys330Glu) variant in the plasminogen gene. Orphanet J Rare Dis.

[53] Dewald G (2018). A missense mutation in the plasminogen gene, within the plasminogen kringle 3 domain, in hereditary angioedema with normal C1 inhibitor. Biochem Biophys Res Commun.

[54] Germenis AE, Loules G, Zamanakou M, Psarros F, Gonzalez-Quevedo T, Speletas M (2018). On the pathogenicity of the plasminogen K330E mutation for hereditary angioedema. Allergy.

[55] Belbezier A, Hardy G, Marlu R, Defendi F, Dumestre-Perard D, Boccon-Gibod I (2018). Plasminogen gene mutation with normal C1 inhibitor hereditary angioedema: three additional French families. Allergy.

[56] Yakushiji H, Hashimura C, Fukuoka K, Kaji A, Miyahara H, Kaname S (2018). A missense mutation of the plasminogen gene in hereditary angioedema with normal C1 inhibitor in Japan. Allergy.

[57] de Maat S, Bjorkqvist J, Suffritti C, Wiesenekker CP, Nagtegaal W, Koekman A (2016). Plasmin is a natural trigger for bradykinin production in patients with hereditary angioedema with factor XII mutations. J Allergy Clin Immunol.

[58] Ivanov I, Matafonov A, Sun MF, Mohammed BM, Cheng Q, Dickeson SK (2019). A mechanism for hereditary angioedema with normal C1 inhibitor: an inhibitory regulatory role for the factor XII heavy chain. Blood.

[59] Maas C (2019). Plasminflammation—an emerging pathway to bradykinin production. Front Immunol.

